# Draft Genome Sequence of *Stenotrophomonas maltophilia* Strain M30, Isolated from a Chronic Pressure Ulcer in an Elderly Patient

**DOI:** 10.1128/genomeA.00576-14

**Published:** 2014-06-12

**Authors:** Pol Huedo, Óscar Conchillo-Solé, Daniel Yero, Sònia Martínez-Servat, Xavier Daura, Isidre Gibert

**Affiliations:** a Institut de Biotecnologia i de Biomedicina, Universitat Autònoma de Barcelona, Barcelona, Spain; b Departament de Genètica i de Microbiologia, Universitat Autònoma de Barcelona, Barcelona, Spain; c Catalan Institution for Research and Advanced Studies (ICREA), Barcelona, Spain

## Abstract

*Stenotrophomonas maltophilia* is an emerging opportunistic pathogen with an increasing prevalence of multidrug-resistant strains. Here, we report the draft genome sequence of *S. maltophilia* strain M30, isolated from a pressure ulcer in an elderly patient.

## GENOME ANNOUNCEMENT

*Stenotrophomonas maltophilia* is an aerobic ubiquitous Gram-negative bacillus commonly isolated from hospital environments (1). Although *S. maltophilia* displays limited invasiveness and pathogenic capacity, it is capable of infecting a wide range of tissues and organs, especially in immunocompromised patients (2). Its disease patterns include bacteremia, catheter-related infections, pneumonia, biliary and urinary tract infections, and skin infections. The therapeutic agent of choice is typically trimethoprim-sulfamethoxazole (3, 4), but resistance to this drug is increasingly being reported (5). New insights into the mechanisms of drug resistance are needed in order to identify new effective drug targets.

*S. maltophilia* strain M30 was isolated from a pressure ulcer of an elderly patient in the Hospital Municipal de Badalona (Barcelona, Spain) in 2009. The M30 strain is a multidrug-resistant (MDR) organism, showing resistance not only to tetracycline, kanamycin, sulfamethoxazole, and erythromycin (6), but also to the complement-mediated bactericidal action of serum (6). Multilocus sequence type analysis (7) revealed that M30 belongs to a new sequence type (sequence type 76 [ST-76]) (6) clustering within a new genetic group (genogroup C) previously described by Kaiser et al. (7). This genetic group comprises clinical isolates from different geographic regions and includes the model MDR strain D457 (8). Recently, it was demonstrated that in strain M30, the diffusible signal factor (DSF)-mediated quorum-sensing system is regulated by a new *rpf* cluster variant (9).

Genomic DNA was extracted with GenElute bacterial genomics DNA kit (Sigma-Aldrich), and whole-genome sequencing was performed using Illumina MiSeq technology at the Universitat Autònoma de Barcelona Genomics core facility. The low-quality reads were filtered, and the remaining reads were *de novo* assembled using VelvetOptimiser version 2.2.5 (10) relaying on Velvet version 1.2.10 (11) and improved with the IMAGE program from the PAGIT package version 1 (12). The assembly resulted in 193 contigs (G+C content, 66.3%), with an *N*_50_ contig size of 46,399 nucleotides, covering a total of 4,902,008 bp. The average length of the contigs is 25.4 kb,nd a the biggest contig contains 142,025 bp.

Genome annotation was performed by the NCBI Prokaryotic Genome Annotation Pipeline version 2.5 (rev. 434060), and 4,515 genes were predicted, of which 4,392 are coding sequences (CDSs), 43 are pseudogenes, 11 are rRNAs (5S, 16S, and 23S), 68 are tRNAs, and 1 is a noncoding RNA (ncRNA). Among the predicted CDSs of M30, we found that 357 genes are not shared with the six strains of *S. maltophilia* (K279a, D457, JV3, R551-3, EPM1, and Ab55555) for which complete genome sequences were available at NCBI (http://www.ncbi.nlm.nih.gov/genome) at the time of analysis. Most of these unique genes encode hypothetical proteins, enzymes related to DNA metabolism and repair, and transposases and integrases, indicating that horizontal gene transfer may be an important source of genomic diversity in *S. maltophilia*. Notably, the set of genes exclusive to M30 also encoded proteins involved in host-microbe interactions, including ankyrin repeat (ANK)-containing proteins (13), two predicted hemolysins, one protease, and one amidohydrolase.

This draft genome will help improve our understanding of genome-associated resistance mechanisms in *S. maltophilia* and the virulence factors the bacterium exploits in order to become a pathogen.

### Nucleotide sequence accession numbers.

This whole-genome shotgun project has been deposited at DDBJ/EMBL/GenBank under the accession no. JELS00000000. The version described in this paper is JELS02000000.
